# Plasma interleukin-7 correlation with human immunodeficiency virus RNA and CD4+ T cell counts, and interleukin-5 with circulating hepatitis B virus DNA may have implications in viral control

**DOI:** 10.3389/fmed.2022.1019230

**Published:** 2022-11-03

**Authors:** Jaisheela Vimali, Yean Kong Yong, Amudhan Murugesan, Kumaran Vishnupriya, Rajeev Ashwin, Evangeline Ann Daniel, Pachamuthu Balakrishnan, Sivadoss Raju, Mohamed Rosmawati, Vijayakumar Velu, Marie Larsson, Esaki M. Shankar

**Affiliations:** ^1^Infection Biology, Department of Biotechnology, Central University of Tamil Nadu, Thiruvarur, India; ^2^Laboratory Centre, Xiamen University Malaysia, Sepang, Selangor, Malaysia; ^3^Department of Microbiology, Government Theni Medical College and Hospital, Theni, India; ^4^Health Center, Central University of Tamil Nadu, Thiruvarur, India; ^5^National Institute for Research in Tuberculosis, Indian Council of Medical Research, Chennai, India; ^6^Department of Microbiology, Centre for Infectious Diseases, Saveetha Dental College and Hospitals, Saveetha Institute of Medical and Technical Sciences (SIMATS), Chennai, India; ^7^State Public Health Laboratory, Directorate of Public Health and Preventive Medicine, Chennai, India; ^8^Division of Gastroenterology and Hepatology, Department of Medicine, Faculty of Medicine, Universiti Malaya Medical Center, Kuala Lumpur, Malaysia; ^9^Division of Microbiology and Immunology, Emory Vaccine Center, Emory National Primate Research Center, Emory University, Atlanta, GA, United States; ^10^Division of Molecular Medicine and Virology, Department of Biomedicine and Clinical Sciences, Linköping University, Linköping, Sweden

**Keywords:** chronic viral hepatitis B, cytokines, hepatitis C, liver enzymes, viral load, HIV

## Abstract

Chronic viral infections represent a leading cause of global morbidity and mortality. Chronic HBV, HCV, and HIV infections result in cytokine perturbations that may hold key implications in understanding the complex disease mechanisms driving virus persistence and/or resolution. Here, we determined the levels of various plasma cytokines using a commercial Bio-Plex Luminex cytokine array in chronic HBV (*n* = 30), HCV (*n* = 15), and HIV (*n* = 40) infections and correlated with corresponding plasma viral loads (PVLs) and liver parameters. We observed differential perturbations in cytokine profiles among the study groups. The cytokines levels positively correlated with PVL and liver transaminases. The monocyte-derived cytokines viz., MIP-1β, IL-8, and TNF-α, and Th2 cytokines like IL-4, IL-5, and IL-13 showed a better correlation with liver enzymes as compared to their corresponding PVLs. Our investigation also identified two cytokines viz., IL-5 and IL-7 that inversely correlated with HBV DNA and HIV PVLs, respectively. Regression analysis adjusted for age showed that every increase of IL-5 by one unit was associated with a reduction in HBV PVL by log_10_ 0.4, whereas, every elevation by a unit of IL-7 was associated with decreased HIV PVL by log_10_ 2.5. We also found that IL-7 levels correlated positively with absolute CD4+ T cell counts in HIV-infected patients. We concluded that plasma IL-5 and IL-7 may likely have a key role on viral control in HBV and HIV infections, respectively. A noteworthy increase in cytokines appears to bear protective and pathological significance, and indeed is reflective of the host’s versatile immune armory against viral persistence.

## Introduction

Viruses have evolved multiple strategies to necessitate chronic infection in spite of the strong antiviral immune sentries operational in the host (1). Continued viral dissemination and consequent disease progression depends on active and consistent release of infectious virions (viral fitness) in the host. The murine lymphocytic choriomeningitis virus (LCMV), the human immunodeficiency virus (HIV), hepatitis B virus (HBV), and hepatitis C virus (HCV) are the predominant chronically-infecting viruses well-studied thus far (2). The pathological impact of viral persistence on the host includes cellular and tissue damage, release of pro-inflammatory mediators, and various cellular and humoral immune perturbation events. Nonetheless, certain chronically-infecting viruses are “quite good” at circumventing the inflammatory signals; for instance, HBV persists for weeks without significantly inducing any immune response as part of its “stealth” strategy to establish chronic infection (3). It is also evident that chronically-infecting viruses recruit a sophisticated regulatory network to usurp the host’s defense sentinels for persistence. Further, in order to control the exuberant proliferation of virus-specific T cells and minimize detrimental immunopathology, the host immune responses need to be adequately regulated (4).

With over two billion people infected with HBV world-wide, ∼350 million are chronically infected, with considerable odds for developing cirrhosis and hepatocellular carcinoma (HCC) (5–7). The hallmark of chronic HBV infection is the failure to mount a coordinated adaptive immune responses to clear the virus. HBV clearance from the liver depends on coordinated innate and adaptive immune responses. Given the non-cytopathic nature of HBV, the liver inflammation and fibrosis that exacerbate long-term chronic infection are primarily driven by the host’s immune responses (7). Akin to HBV, most acute HCV infections largely remain asymptomatic. However, in the absence of treatment, ∼30% of the infected individuals spontaneously achieve viral clearance within 6 months, whereas, ∼70% progress to develop chronic liver disease. Cirrhosis can develop in ∼15–30% of those with persistent HCV infection within 20 years (8). HCV is able to evade a plethora of immunological arsenals, including cytokine responses to achieve a chronic disease state (9). A coordinated and efficient immune response is rendered by an intricate network of cytokines during the initial phases of infection. Nonetheless, HCV is largely successful in immune evasion by engineering a unique cytokine profile, which interferes with viral clearance at multiple levels. HIV represents yet another key chronic infection that continues to pose a serious global public health threat causing over 36.3 million deaths till date. By 2020, it was predicted that there were 37.7 million HIV-infected individuals worldwide. Chronic HIV infection results in detrimental loss to CD4+ T helper cells and eventually to the overall immune functions that are primarily orchestrated by the cells (10).

Cytokines are a diverse group of molecules that play a critical role in host immune responses. T helper 1 (Th1) responses marked by interleukin-2 (IL-2), gamma interferon (IFN-γ); Th2 responses heralded by signature cytokines IL-4, IL-5, IL-13, and IL-31, and Th17 cytokines (e.g., IL-17, IL-22, IL-23), are essential in mediating inflammation and antiviral surveillance (5, 11). Cytokines play significant roles in the defense against viral infections by binding to their cognate receptors expressed on target cells and initiating downstream signaling pathways (12). Evidence suggest that the prognosis of HBV infection may be affected by altered cytokine expression in chronic HBV-infected patients (13). HBV-specific IFN-producing T cells are implicated with viral clearance, whereas, HBV-specific TNF-α producing T cells are associated with liver pathology (14). HBV and HCV exhibit remarkably different patterns of cytokines despite that both conditions are caused by viruses leading to chronic inflammation (15). Their immunopathogenesis is influenced by a perturbation in pro-inflammatory and anti-inflammatory cytokine homeostasis (16). The chronic nature of HCV infection may be attributed to a skewed Th2 response, whereas, HIV infection results in dysregulated expression of a variety of bioactive cytokines (17). The impact of PVL would likely be influenced by cytokine perturbations in chronic viral infections (18, 19). Determining the perturbations in cytokine profiles during viral infections is crucial for understanding the mechanisms driving virus persistence or resolution of infection (20). Here, we determined the levels of various cytokines in chronic HBV, HCV, and HIV infections for arriving at necessary correlations with their PVLs and pathological liver parameters.

## Materials and methods

### Ethical approval

The cross-sectional cohort investigation was conducted after the Institutional Ethical Committee (IEC) of the Government Medical College, Theni, India (Ref. No. 2544/ME1/18 and Ref. No. 1515/MEIII/21) approved the study protocols involving human participants. The host institution’s Institutional Biosafety Committee (IBSC) approval was also obtained for the conduct of viral hepatitis investigations (Ref. No.: CUTN/SLS/1st IBSC/2020/04). The research was carried out in compliance with good clinical practice, including the International Conference on Harmonization Guidelines and the Declaration of Helsinki. All the human participants or their legal representatives who provided written informed consent to participate in the program. The institutional ethical committee (IEC) approved the written consent form, which was signed by the respective participants and a copy of which was provided to the subject for records.

### Study subjects

**FIGURE 1:**
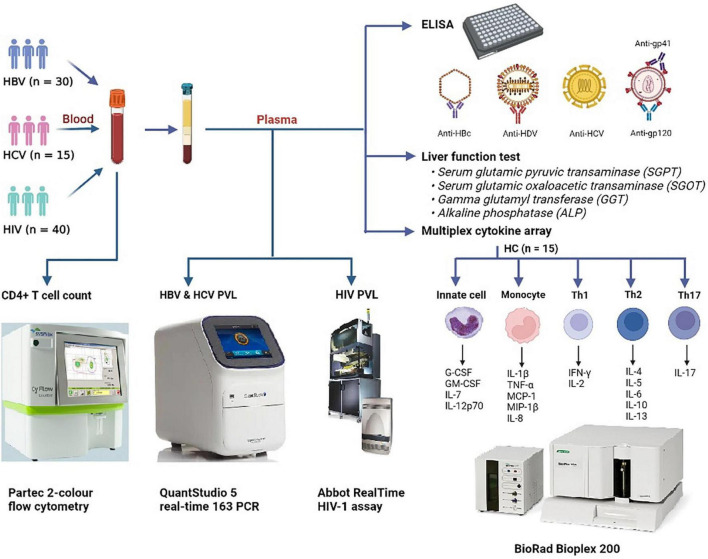
Graphical outline of the study. Peripheral blood was collected from patients with HBV (*N* = 30), HCV (*N* = 15), and HIV (*N* = 40) and were subjected to CD4+ T cell counts and HIV, HBV, and HCV viral load estimations. Anti-HBc IgG, anti-HDV IgM, anti-HCV, and anti-HIV-1/2 antibodies were screened by ELISA. Liver function test was performed to estimate the levels of SGPT, SGOT, GGT, and ALP in plasma. Plasma cytokine levels were determined by a commercial Bio-plex cytokine array. HBV, hepatitis B virus; HCV, hepatitis C virus; HDV, hepatitis delta virus; HIV, human immunodeficiency virus; HC, healthy control; SGOT, serum glutamic oxaloacetic transaminase; SGPT, serum glutamic-pyruvic transaminase; GGT, gamma-glutamyl transferase; ALP, alkaline phosphatase.

The study included a biologically defined male and female subjects with age ≥18 years. A total of 40 HIV-infected individuals [as per the criteria of the National AIDS Control Organization (NACO), India], 30 HBV-infected subjects with HBsAg positivity, 15 HCV-infected people with anti-HCV positivity, and 16 healthy controls (HCs) were recruited in the investigation. None of the HBV- and HCV-infected individuals were on treatment with antiviral agents (viz., sofosbuvir and/or velpatasvir for HCV), including IFN-α or ribavirin and/or any immunosuppressive medications (i.e., systemic steroids/anti-inflammatory drugs). HCs were defined as subjects free of pulmonary tuberculosis and chronic viral illnesses (HBV, HCV, and HIV). A skilled phlebotomist from the Government Medical College, Theni’s Hepatology Unit collected peripheral blood from all the infected participants and healthy controls. The summary of the work is presented in Figure 1.

### Liver function test

All patients and HCs had their liver biochemical markers examined. Using a semi-automatic analyzer (Rapid Diagnostics Star 20, Hyderabad, India), the plasma levels of serum glutamic oxaloacetic transaminase (SGOT), serum glutamic-pyruvic transaminase (SGPT), gamma glutamyl transferase (GGT), and alkaline phosphatase (ALP) were measured using a commercial kit (TRUEchemie, Brussels, Belgium) SGOT assay kit (Lot. No. A1921102) with the cut-off values set at 33U/L, SGPT assay kit (Lot. No. A1521101) with the cut-off values set at 35U/L, GGT assay kit (Lot. No. G1221122), with the cut-off values set at 49U/L (male) and 32U/L (female), ALP assay kit (Lot. No. A1321111) with the cut-off values set at 141U/L, respectively.

### Human immunodeficiency virus diagnosis and absolute CD4+ T cell count

Human immunodeficiency virus infection was diagnosed using a standard three kit strategy as recommended by the NACO, India. HIV-1 diagnosis was based on rapid immunochromatographic tests viz., Comb Aids-RS (Arkray Healthcare, Mumbai, India), VoXpress HIV-1/2 (Voxtur Bio, Mumbai, India), and Meriscreen HIV 1-2 WB (Meril Life, Chennai, India) for detection of antibodies specific to gp41 and gp120 of HIV -1 and gp36 of HIV -2 in human serum, plasma and whole blood, as per the instructions of the manufacturers. All HIV infected patients were on highly active antiretroviral therapy (HAART) at the time of inclusion into the investigation. For absolute CD4+ T-cell enumeration, 2 ml of whole blood in K_3_EDTA tubes was collected for processing within 6 h, and was performed using the Partec two-color flow cytometer (Sysmex, Germany) anti-CD45-PE-Cy5 (Cat. No. 05-8405-02) and anti-CD4-PE (Cat. No. 05-8405-01) fluorochrome tagged antibodies were used for the immunophenotyping as per the manufacturer’s instructions.

### Plasma viral load for human immunodeficiency virus, hepatitis B virus, and hepatitis C virus

Hepatitis B virus and HCV viral loads were estimated using the Pathodetect™ HCV quantitative Real-Time PCR kit (Mylab Discovery Solutions, Pune, India). The Myolab Discovery Solutions Pathodetect™ HBV quantitative PCR kit was employed to quantify HBV DNA and HCV RNA through *in vitro* nucleic acid amplification test. Viral load quantitation was performed on a QuantStudio 5 real-time PCR systems (Applied Biosystems, ThermoFisher Scientific, Waltham, MA, USA). The test can detect and quantify HBV DNA over the range of 10–1 × 10^8^ IU/ml with the plasma sample volume of 200 μl. The sensitivity of the test for HCV detection and quantification was 40–7 × 10^7^ IU/ml for the sample volume of 200 μl. HIV PVL was determined using the Abbott RealTime HIV-1 assay (Abbott, IL, USA), an *in vitro* reverse transcription-polymerase chain reaction (RT-PCR) assay for the quantitation of HIV-1 in the plasma of HIV-infected individuals. The sensitivity of the test was 40 copies/ml for 1 ml sample volume.

### Enzyme-linked immunosorbent assay

The expression of anti-hepatitis B core antigen (anti−HBc) (Dia. Pro, Milano, Italy, BCAB.CE. Lot. No, C10T14/3), and anti-hepatitis D virus IgM (Dia. Pro, Milano, Italy, HSN-3822) in the patients’ plasma were determined. All the readings were measured using an ELISA multimode microplate reader (BioRad, Hercules, CA, USA).

### Proximity ligation assay

A commercial ProQuantum™ Human IL-21 Immunoassay kit (ThermoFisher Scientific, USA) that uses the proximity ligation assay technology was employed to determine the levels of IL-21 in plasma on a Prism 7900HT (Applied Biosystems, Foster City, CA, USA) qRT-PCR platform in 96-well optical fast-plates as per the manufacturer’s instructions.

### Bio-plex luminex cytokine array

Bio-plex Pro Human Cytokine Grp I Panel 17-plex kit (BioRad, Hercules, CA, USA) was used to quantify the following plasma cytokines such as, MCP-1, G-CSF (filgrastim), GM-CSF, IL-7, IL-12(p70), IL-1β, MIP-1β, TNF-α, CXCL8 (IL-8), IFN-γ, IL-6, IL-2, IL-4, IL-5, IL-13, IL-17, and IL-10, according to the manufacturer’s instructions (Supplementary Table 1). Briefly, the samples were prepared by diluting 20 μl of plasma with 80 μl of Bio-plex diluent. The cytokine standard was diluted in standard diluent on ice followed by 4-fold repeated dilutions. The 10× bead (570 μl) was diluted in 5,130 μl of assay buffer in the dark, and 50 μl of the preparation was added to all the wells, followed by the same volume of blank, standard, and sample, and incubated for 30 min at 850 rpm. Meanwhile, 300 μl of detection antibody was diluted in 2,700 μl of diluent and 25 μl of the solution was added to each well, followed by 30 min of incubation at 850 rpm. Streptavidin PE was prepared by diluting 60 μl of SAPE with 5,940 μl of assay buffer. The solution was vortexed, and then 50 μl was added to each well, followed by a 10 min incubation at 850 rpm. The 125 μl of assay buffer was resuspended to all the wells and incubated at 850 rpm for 30 s. Finally, the plate was placed on a Bio-plex micro-plate platform for cytokine quantification, and data with a coefficient of variability (CV) <10% were included. The Bio-plex Manager software was used for standard curve optimization and cytokine estimation.

### Statistical analysis

**FIGURE 2:**
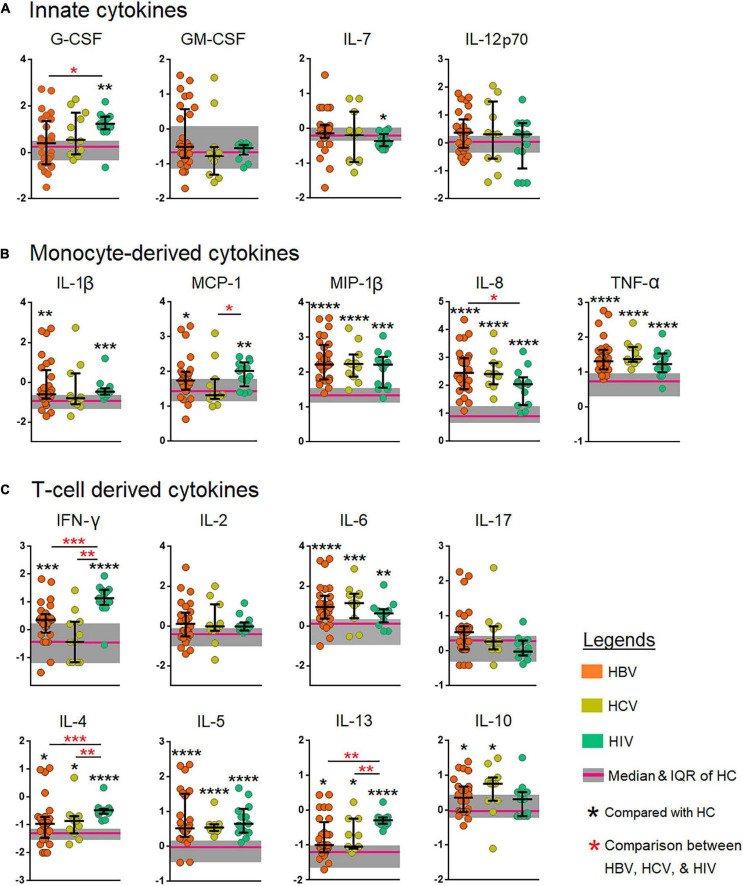
Comparison of the levels of cytokines among patients chronically infected with hepatitis B virus (HBV), hepatitis C virus (HCV), and human immunodeficiency virus (HIV). **(A)** Innate cytokines **(B)** Monocyte-derived cytokines, and **(C)** T cell-derived cytokines. The cytokines were compared across the three patient groups by the Kruskal–Wallis test. *Post-hoc* Mann–Whitney U tests were subsequently performed only for those biomarkers with a Kruskal–Wallis test *p*-value of <0.05. *p*-values <0.05 are considered significant; *p < 0.05, **p < 0.01, ***p < 0.001, ****p < 0.0001. G-CSF, granulocyte-colony stimulating factor; GM-CSF, granulocyte-macrophage colony-stimulating factor; IFN-γ, interferon gamma; MCP-1, monocyte chemoattractant protein-1; MIP-1β, macrophage inflammatory protein-1 beta; TNF-α, tumor necrosis factor alpha; IL, interleukin.

**FIGURE 3:**
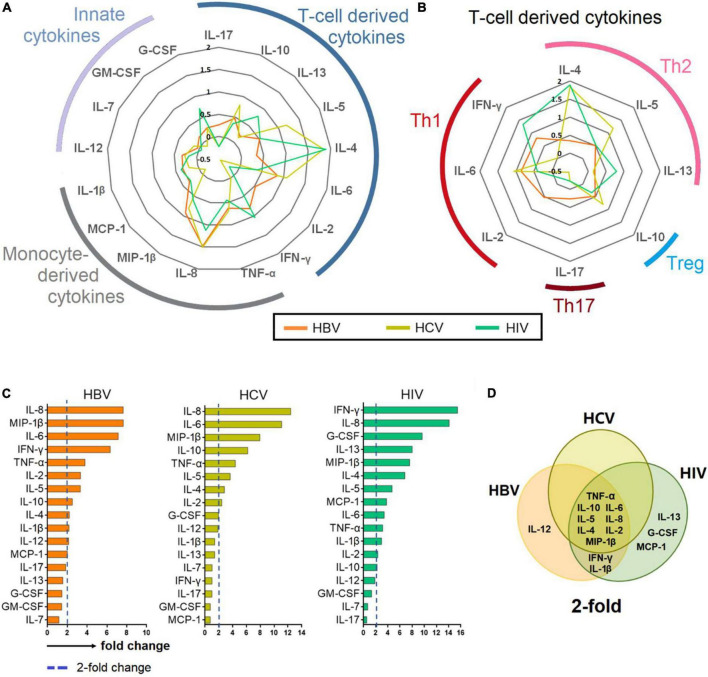
Cytokine profiling in chronic hepatitis B virus (HBV), hepatitis C virus (HCV), and human immunodeficiency virus (HIV) infections. **(A–B)** Cytokines with a > 2-fold change in patients chronically infected with HBV, HCV and HIV normalized against HCs. **(C)** Bar plot showing mean 2-fold change among all cytokines ranked in descending order. **(D)** Venn diagram depicting cytokines that are upregulated >2-fold. The Venn diagram identified 8 cytokines that are common among patients chronically infected with HBV, HCV and HIV.

**FIGURE 4:**
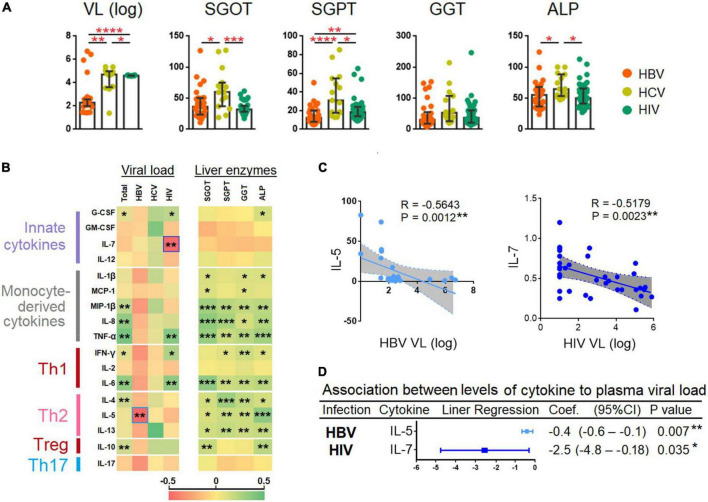
Correlation between plasma viral loads, levels of liver transaminases, and cytokines. **(A)** Comparison between plasma viral loads (PVL) and liver enzymes among patients chronically infected with HBV, HCV, and HIV. The cytokines were compared across the three patient groups by a Kruskal–Wallis test. *Post-hoc* Mann–Whitney U tests were subsequently performed for those biomarkers with a Kruskal–Wallis test *p*-value of <0.05. **(B)** Spearman correlation of viral load and liver enzymes and the 17 cytokines. The R-value of each comparison was reflected by the color scale of the heatmap. **(C)** Spearman correlation of the two cytokines that had an inverse correlation with PVL. The exact R- and *P*-values were calculated. **(D)** Association between the levels of cytokines with PVL. *P*-values < 0.05 are considered significant in all tests; *p < 0.05, **p < 0.01, ***p < 0.001, ****p < 0.0001. HBV, hepatitis B virus; HCV, hepatitis C virus; HIV, human immunodeficiency virus; HC, healthy control; SGOT, serum glutamic oxaloacetic transaminase; SGPT, serum glutamic-pyruvic transaminase; GGT, gamma-glutamyl transferase; ALP, alkaline phosphatase; VL, viral load.

The aim of the investigation was to identify cytokine profiles associated with anti-viral responses. The demographic and clinical characteristics of study groups (viz., HBV, HCV, HIV, and HCs) were compared. Comparisons of categorical variables were done using the Chi-square, whilst continuous variables, using a non-parametric Kruskal–Wallis or Mann-Whitney U test as appropriate. The levels of cytokines, PVLs and liver enzymes between the three infection groups were done using the Kruskal–Wallis test. If the *p*-values were <0.05, three-way comparisons were performed using the Mann-Whitney U test across the three infected groups. The cytokine levels between each infection groups were compared with the HCs using the Mann-Whitney U test (Figures 2, 4A). The cytokine fold-change was calculated by normalizing the respective cytokine against the median of the same cytokine in the HC group. The fold-change of each infection group was then ranked in a descending order (Figure 3C). Cytokines that upregulated >2-fold from each infection group were used to produce a Venn diagram (Figure 3D) in order to identify the cytokines involved in anti-viral responses. Continuous variables such as PVL, liver enzyme and cytokines levels were compared using the Spearman correlation and displayed as heatmap (Figure 4B), and the cytokines that correlated inversely with the PVL were identified (Figure 4C). The association between these cytokines and the PVL were evaluated using a linear logistic regression model controlling for age. The coefficient and 95% confidence interval (CI) were estimated (Figure 4D). The analyses were performed using GraphPad PRISM, ver.5.02 (GraphPad Software, San Diego, CA, USA). Linear regression was performed using SPSS, SPSS ver.24.0 (SPSS Inc., Chicago, IL, USA). Two-tailed *p* < 0.05 was considered as statistical significance for all the tests performed. *p*-values < 0.05, <0.01, <0.001, <0.0001 were marked as *, **, ***, ****, respectively.

## Results

### Clinico-demographic features and serological diagnosis

The four-group, non-randomized trial design included samples from 30 (20 males and 10 females) subjects with chronic HBV infection (Group 1), 15 (eight males and seven females) individuals with chronic HCV infection (Group 2), 40 (21 males and 19 females) subjects with HIV infection on HAART (Group 3) and 16 (eight males and eight females) healthy controls (Group 4). The specimens were collected between September and October 2021. All HBV-infected individuals, 20% HCV-infected individuals, and 15% HIV-infected patients were positive for anti-HBc. The detection of anti-HDV IgM in all the HBV-infected samples was dismissive. The human IL-21 immunoassay revealed negligible plasma levels of IL-21. The clinico-demographic and laboratory characteristics of the cohort are summarized in Table 1.

**TABLE 1:** Clinicodemographic characteristics of study participants.

Characteristic	Total (*n* = 101)	HBV (*n* = 30)	HCV (*n* = 15)	HIV (*n* = 40)	HC (*n* = 16)	*P*-value
Age, year; median (IQR)	42 (27.5–53.5)	42.5 (30.2–64)	52 (46–61)	44 (32.5–51.5)	29 (19–42)	<0.0001****
Sex, male; *n* (%)	54 (53.5)	19 (63.3)	8 (53.3)	20 (50)	7 (43.7)	0.578
HDV seropositivity, *n* (%)	30 (29.7)	0 (0)	…	…	…	…
Viral load, log; copies/ml	…	2.25 (1.82–2.57)	4.68 (3.47–4.93)	4.6 (4.59–4.63)	…	…
**Liver enzymes, median (IQR)**						
SGOT, U/L; median (IQR)	14.6 (8.3–22.5)^†^	36.2 (23.6–50.4)	60 (37.1–75)	32 (28.3–37.7)	…	0.0032**
SGPT, U/L; median (IQR)	33.1 (16.4–57.3)^†^	12.4 (8.0–20.3)	31.1 (17.6–54.6)	18.4 (13.9–24.1)	…	<0.0001****
GGT, U/L; median (IQR)	49.8 (34.4–65.3)^†^	30.7 (17.9–55.6)	53.3 (26–107.5)	36.9 (20.7–61.1)	…	0.0879
ALP, U/L; median (IQR)	49.8 (34.4–65.3)^†^	55.3 (36.9–68.2)	64.7 (53.7–88.5)	50.3 (41.3–65.7)	…	0.0413
CD4+ T cell count, cells/μl	…	…	…	211 (119–259)	…	…

All data reported as numbers (*n*) and percentages (%) unless specified. %, percentage; IQR, interquartile range; HBV, hepatitis B virus; HCV, hepatitis C virus; HIV, human immunodeficiency virus; HC, healthy control; SGOT, serum glutamic oxaloacetic transaminase; SGPT, serum glutamic-pyruvic transaminase; GGT, gamma-glutamyl transferase; ALP, alkaline phosphatase.

*, **, ***, **** represent *p* < 0.05, <0.01, <0.001, and <0.0001, respectively.

^†^The median (IQR) was calculated based on HBV, HCV, and HIV patients only, *n* = 85.

The clinical and liver biochemical parameters suggested that 56% (*n* = 17) of HBV-infected individuals, 80% (*n* = 12) HCV-infected individuals, 47.5% (*n* = 19) HIV-infected individuals were classified as having chronic hepatitis and 43% (*n* = 13) of HBV-infected individuals, 20% (*n* = 3) HCV-infected individuals, and 52.5% (*n* = 21) HIV-infected individuals were chronically infected without any underlying clinical and/or laboratory evidence of hepatic injury.

### Profiling of innate plasma cytokine levels in human immunodeficiency virus showed significantly elevated levels of interleukin-7 and granulocyte-colony stimulating factor but not granulocyte-macrophage colony-stimulating factor and interleukin-12

We analyzed the plasma levels of four innate cytokines viz., G-CSF (filgrastim), GM-CSF, IL-7, and IL-12 (p70) across the four study groups using the Kruskal-Wallis and Mann-Whitney (Figure 2A) tests. G-CSF was found to be significantly high among HIV-infected individuals (*p* < 0.01) in comparison with the HCs. G-CSF was also significantly elevated among HIV-infected individuals as compared to the HCV-infected individuals. IL-7 was significant only among HIV-infected individuals (*p* < 0.05). GM-CSF and IL-12 were not significant in any of the infected individuals. This suggests that chronic HIV infection likely leads to increased release of G-CSF in the host.

### Monocyte-derived cytokines in the plasma revealed differential profiling among human immunodeficiency virus, hepatitis B virus, and hepatitis C virus-infected individuals

Next, we profiled the monocyte-derived cytokines such as IL-1β, MCP-1, MIP-1β, TNF-α, and IL-8 in the plasma of the four study groups using the Kruskal-Wallis test (Figure 2B). MCP-1 was found to be significantly high among HBV- and HIV-infected individuals with *p* < 0.05 and *p* < 0.01, respectively when compared to HCs. In a comparison between HBV-, HCV-, and HIV-infected individuals MCP-1 was found to be significantly increased (*p* < 0.01) when compared among all the three infected groups. A significant increase in plasma MIP-1β (*p* < 0.0001) was found in HBV-, HCV-infected individuals, and among HIV-infected individuals (*p* < 0.001). In the case of TNF-α, the HBV-, HCV- and, HIV-infected individuals showed a high level of significance (*p* < 0.0001) than HCs. IL-8 was found to be highly significant across all the three infected groups with *p* < 0.0001 with HCV at the highest significance range. The level of IL-1β was significantly high among the HIV (*p* < 0.001) and among chronic HBV-infected individuals (*p* < 0.01).

### Differential expression of Th1, Th2, Treg, and Th17 cytokines among the various chronically-infected study groups

T helper 1 cytokines (IFN-γ, IL-6, and IL-2), Th2 cytokines (IL-4, IL-5, and IL-13), Th17 cytokine (IL-17) and the Treg signature cytokine IL-10 were measured in the plasma of the four distinct groups of our study cohort (Figure 2C). IFN-γ was highly significant among HIV-infected individuals (*p* < 0.0001) and was significantly increased in HBV-infected individuals (*p* < 0.001). No significance was found in HCV individuals in comparison with HCs. We also noticed a significantly increased abundance in IL-6 levels among HBV- and HIV-infected individuals with *p* < 0.01 and *p* < 0.001, respectively. No statistically significant increase was observed in IL-2 levels across any of the infected study groups.

Interleukin-4 was found to be highly significant among HIV individuals (*p* < 0.0001) and among HBV- and HCV-infected individuals (*p* < 0.05) in comparison with HCs. When compared among the infected groups, IL-4 levels of HBV and HCV were significantly higher with *p* < 0.001 and *p* < 0.01, respectively. IL-5 were found to be significantly increased among all the infected groups (*p* < 0.0001). IL-6 was significantly raised in HBV (*p* < 0.0001) > HCV (*p* < 0.001) > HIV (*p* < 0.01) > HCs. IL-10 was significantly high among HBV- as well as HCV-infected individuals (*p* < 0.05) relative to HCs. IL-13 was significantly increased among HIV (*p* < 0.0001) > HBV (*p* < 0.05) = HCV (*p* < 0.05) as compared with HCs. A comparison among all the infected individuals revealed that HBV and HCV resulted in significantly increased levels of IL-13 (*p* < 0.01) than in HIV-infected individuals.

### Cytokine elevations in chronic hepatitis B virus, hepatitis C virus, and human immunodeficiency virus infections were suggestive of T cell and monocyte-derived and had a Th2 skew

The fold change of cytokines was determined by normalizing the level of each cytokine against the median level of the respective cytokine in the HC group. The median levels of cytokines among HBV-, HCV-, and HIV-infected patients were then presented as a radar chart. Our analysis showed that the infected groups had a common profile where the elevated cytokines were predominantly T cell and monocyte-derived and with a Th2 bias (Figures 3A,B).

The fold change of the cytokines was than ranked in descending order, and those cytokines that elevated greater than 2-fold for each chronic infections were identified and presented in Venn-diagram (Figures 3C,D). The analysis showed that among all the 17 cytokines, eight viz., TNF-α, IL-10, IL-6, IL-5, IL-8, IL-4, IL-2, MIP-1β were common among patients chronically infected with HBV, HCV, and HIV. IFN-γ and IL-1β were common among chronically infected with HBV and HIV.

### Plasma interleukin-5 and interleukin-7 levels were inversely correlated with hepatitis B virus and human immunodeficiency virus plasma viral loads

Plasma viral loads quantification was performed across all the HBV-, HCV-, and HIV-infected individuals. Using the Kruskal–Wallis and the Mann-Whitney U tests, we found a significant increase in HBV PVLs (*p* < 0.0001) > HCV (*p* < 0.01) > HIV (*p* < 0.05) (Figure 4A). We also performed a Spearman correlation analysis and results are presented as a heat map (see Figure 4B). Our results showed that the cytokines levels were positively correlated to PVL (combination of HBV, HCV, and HIV PVLs) and liver enzymes viz., SGOT, SGPT, GGT, and ALP. The cytokines showed a better correlation with liver enzymes as compared to PVL, particularly with monocyte-derived cytokines (viz. MIP-1β, IL-8, and TNF-α) and Th2 cytokines viz., IL-4, IL-5, and IL-13 (Figure 4B). The analysis also identified two cytokines viz., IL-5 and IL-7 that inversely correlated with HBV (R = −0.5643 and *p* = 0.0012) and HIV PVL (R = −0.5179, *p* = 0.0023), respectively (Figure 4C).

Regression analysis adjusted for age showed that with every increased of IL-5 by one unit was associated with a reduction in HBV PVL by log_10_ 0.4 (95% CI = −0.6 to −0.1); *p* = 0.007; whereas every increase of IL-7 by one unit was associated with decreased HIV PVL by log_10_ 2.5 (95% CI = −4.8 to −0.18); *p* = 0.035 (Figure 4D).

### CD4+ T cell counts of human immunodeficiency virus-infected individuals correlated with interferon gamma and interleukin-7 levels

**FIGURE 5:**
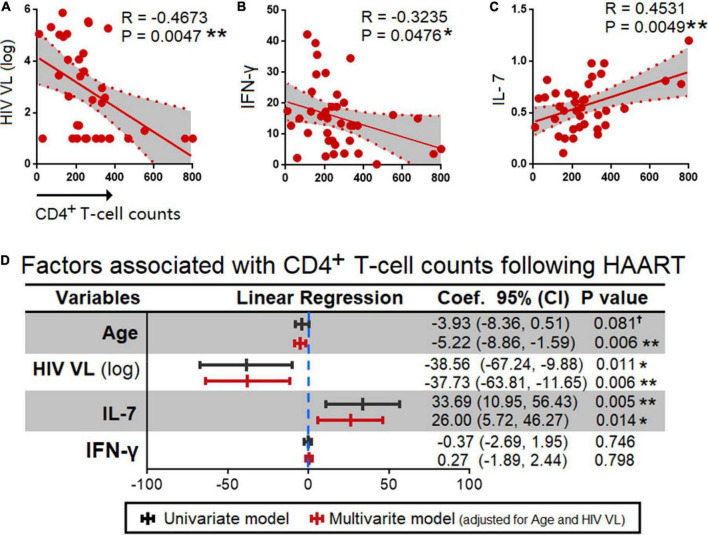
Correlation between HIV plasma viral loads, and cytokines. Spearman correlation **(A)** HIV PVL and **(B)** IFN-γ that had an inverse correlation and **(C)** IL-7 that had a positive correlation with CD4+ T cell counts. The exact R- and *P*-values were calculated. **(D)** Association between age, HIV viral load (log), IL-7, and IFN-γ with CD4+ T cell counts. *P*-values < 0.05 are considered significant in all tests; *p < 0.05, **p < 0.01. HAART, highly active anti-retroviral therapy; HIV, human immunodeficiency virus; VL, viral load, ^†^, having a trend of significance.

Given that, HIV viral load is known for its inverse correlation with the CD4+ T-cell counts (shown by other studies and here in our study, Figure 5A; 21–23), here we investigated the correlation between the 17 cytokines and CD4+ T cell counts (Supplementary Figure 1). The analyses showed that the CD4+ T-cell counts in HIV infected individuals following HAART were inversely correlated with IFN-γ (R = −0.3235, *P* = 0.0476) and IL-7 (R = 0.4531, *P* = 0.0049) (Figures 5B,C).

Next, we performed multivariate and univariate linear regression analyses adjusted for age and HIV viral load, and found that age associated with CD4+ T cell counts was −3.93 (95% CI = −8.36,0.51); *p* = 0.081, −5.22 (95% CI = −8.86, −1.59); *p* = 0.006 by univariant and multivariant regression analyses, respectively. HIV PVL by log 10 was −38.56 (95% CI = −67.24, −9.88); *p* = 0.011, −37.73 (95% CI = −63.81, −11.65); *p* = 0.006 by univariant and multivariant analyses, respectively. Further, in the case of IFN-γ−0.37 (95% CI = −2.69, 1.95); *p* = 0.746, 0.27 (95% CI = −1.89, 2.44); *p* = 0.798 by a univariant and multivariant analyses, respectively. IL-7 was found to be 33.69 (95% CI = 10.95, 56.43); *p* = 0.005, 26.00 (5.72, 46.27); *p* = 0.014 by a univariant and multivariant analysis, respectively (Figure 5D).

## Discussion

Cytokines are critical mediators of virus elimination, and regulators of inflammation leading to optimization of protective responses. Cytokines are also involved in various pathophysiological processes involving the liver that develop following an infection viz., regeneration, and fibrosis (24). Understanding the interactions between infection and cytokine profiling offers an opportunity to understand viral pathogenesis and control. In order to better characterize the immune profile, we classified cytokines into innate, monocyte-derived and T cell-derived. The T-cell derived cytokines were further classified into four sub-classes viz., Th1, Th2, Treg, and Th17. Here, we showed that the cytokines among individuals chronically infected with HBV, HCV, and HIV were suggestive of a preponderance toward Th2 and monocytic polarization. Significant elevation of TNF-α, IL-10, IL-4, IL-5, IL-2, IL-6, IL-8, and MIP-1β were observed among HBV, HCV, and HIV infections in common. The elevation of many cytokines had a better correlation with liver enzymes as compared to viral loads indicating that generalized immune activation might have been induced by damage-associated molecular patterns (DAMPs) rather than by pathogen-associated molecular patterns (PAMPs). Furthermore, we also identified that the levels of IL-5 and IL-7 were inversely correlated with HBV and HIV viral loads, respectively. Strikingly, the IL-7 level was also an independent predictor for higher CD4+ T cell counts in HIV-infected individuals following HAART.

During acute infection, both the host immune response and virus are in constant competition until either the infection killing the host, or the resolution of infection, or it becomes chronic (1). However, during chronic infection viruses develop diverse tactics to subvert host immune surveillance including evasion of pattern recognition receptors (PRRs) (25), modulation of autophagy (26, 27), and even deceiving cell death to their advantage (28, 29). Through a dynamic process, both host and virus activities balance each other where the virus persists without causing any overt disease in the host (1). Given that cytokines are the central mediators of inflammatory responses, it is of paramount importance to identify their profiles associated with persistent viral infection as well as viral elimination. Tissue damage inflicted by virtue of trauma, pathogens or cells dying from necroptosis or pyroptosis, results in the release of PAMPs or DAMPs into the systemic circulation, which triggers inflammation. On control of the acute injury, macrophages suppress inflammation and initiate wound repair by clearing the debris, and produce growth factors and other anti-inflammatory mediators (30).

Liver enzymes (SGOT, SGPT, GGT, and ALP) are produced and held within hepatocytes for aiding in digestion. In a healthy liver where the hepatocyte turnover is minimal, the level of liver enzymes in peripheral blood are also minimal. The perturbation in the specific hepatic enzymes like ALP, SGPT, SGOT, GGT, bilirubin, and albumin (31) in peripheral blood is indicative of hepatocyte death (32), and hence is a useful marker of hepatic disease severity. Chronic hepatitis may cause the plasma SGPT to rise significantly because of their release into the peripheral blood during hepatocellular injury. At times, the SGPT:SGOT ratio can also characterize the pattern of liver disease (33) offering opportunities to draw valuable conclusions on disease diagnosis (34).

Here, we showed that the three chronic viral infections had a common cytokine profile that skewed predominantly toward monocyte-derived as well as Th2 polarization. Furthermore, IL-1β, MIP-1β, MCP-1, IL-8 (monocyte-derived) as well as IL-4, IL-5, and IL-13 (Th2 cytokines) correlated with the levels of all the four liver enzymes suggesting that their elevation was in response to DAMPs released by injured hepatocytes. Of note, IL-1β is a highly potent cytokine produced *via* the inflammasome/caspase-1 pathway and is key to viral elimination (35). IL-1β is produced as biologically inactive forms, pro–IL-1β, and are processed by caspase-1 to biologically active IL-1β *via* NLRP3 activation (36). In addition to processing IL-1β and IL-18, casp1 also mediates the rupture of plasma membrane (37), resulting in the release of inflammatory cellular contents (DAMPs) into the systemic circulation via pyroptosis (38, 39). HBV causes IL-1β attrition by inhibiting the NFκ-B and inflammasome pathways (40) although our study reported an increase in IL-1β levels. HIV patients exhibit a faster rate of fibrosis advancement (41), and the surged levels of IL-1β in chronic HCV infection appears to augment the onset of hepatocellular carcinoma (HCC) (42). A significant increase in IL-1β in chronic HIV infection regulates the rate of chronicity under inflammatory conditions. MCP-1 is a potent chemotactic factor for monocytes (43), driven by IL-1β, IL-6, and TNF-α (44). The importance of MCP-1 is crucial given the protracted course of HBV infection, and provides clues for illuminating the molecular mechanisms underlying viral clearance (45). MCP-1 may be employed as a serological marker for non-invasive assessment of hepatic inflammation in CHB patients (46), and hence assumes significance in the context of HBV chronicity.

Interferon gamma levels appear to be persistently high and is varied among patients with chronic infections (47). Many HIV-derived proteins can directly activate T cells to render IFN-γ synthesis, which causes long-term immune activity, eventually exhausting the cell population causing the cessation of IFN-γ production (48). HIV-infected individuals with high levels of circulating IFN-γ showed slow CD4+ cell recovery after initiating HAART, which likely could be an outcome of interminable immune activation progressing toward immune exhaustion.

Interleukin-7 was reported as a requisite factor for T-cell development (49). HIV infection masked the proliferative effects of IL-7 displaying an inverse correlation between IL-7 plasma levels and CD4+ T cell counts (50). Through persistent T-cell activation, IL-7 may increase viral load (51). We assessed the association between IL-7 levels and CD4+ T cell counts in HIV-infected individuals on HAART. Due to IL-7’s strong ability to increase T-lymphopoiesis, our result possibly supports T-cell immunological reconstitution in conjunction with HAART. IL-7 enhances cytotoxic T cell responses by promoting CD8+ T cell proliferation, activity, and survival. The effects of IL-7 on CD8+ T cells were enhanced by IL-6 during persistent HCV infection (52). Individuals with chronic HCV infection had considerably lower IL-7 levels, which were inversely linked with HCV RNA levels (53). Treatment with IL-7 appears to reduce the rate of progression of HIV, HBV, and HCV viral infections (54). In HIV-infected individuals on ART, IL-7 boosts T cell memory and *de novo* renewal of naive T cells (55). The negative correlation between plasma IL-7 and CD4+ T cell counts suggests either a feedback mechanism for increasing peripheral T cell numbers in lymphopenic hosts (50, 51, 56). Moreover, the plasma IL-7 serves as a promising indicator of CD4+ T cell recovery during treatment (57–59). The impact of IL-7 on T-cell responses might be severely limited by the down-regulation of the IL-7R brought on by high viral load. The increased levels of IL-7 observed in our investigation holds significance in the context of HIV infection where we have found its inverse correlation with PVL. Higher viral load appears to augment STAT1 phosphorylation in response to IL-7 in HIV under *in vitro* conditions (43). The therapeutic potential of IL-7 in the management of chronic viral illnesses also has been deliberated (44) because exogenous administration of IL-7 appears to have restored peripheral mucosal-associated invariant T (MAIT) cell frequencies in HIV-infected individuals on antiretroviral therapy (ART) (60). IL-7 has also been correlated with T-cell depletion, and hence it is possible to assume that the cytokine is crucial for restoring T-cell homeostasis in HIV infection (61). In regards to HBV, IL-7 released by inflamed hepatocytes regulates T-cell activation to enhance the production of Th1 cytokines and IL-17A, which likely aid in HBV clearance (40). Hence, it is likely that the observed increase in IL-7 may have implications with control of viral infections.

Granulocyte-colony stimulating factor are primarily produced by monocytes, fibroblasts and endothelial cells of the stroma of the bone marrow (62). G-CSF can encourage monocytes to convert to an anti-inflammatory/pro-restorative phenotype (M2-like) (63), and could aid in prompt clearance of HBV (64) be inferred that G-CSF may aid in viral clearance during chronic infections. Myeloid lineage cells need GM-CSF in order to proliferate, differentiate, and survive (65). For over 20 years, GM-CSF has been employed in therapeutic settings (66), and poor levels of GM-CSF likely favor pathogenic proliferation in the host (67). Patients with liver illnesses reportedly have higher GM-CSF levels, and may be crucial for both host defense and disease progression (68). GM-CSF is suggestive of pro-inflammatory polarization in HCV patients with cirrhosis (69). A decrease in GM-CSF reflects weakened proliferation and differentiation of the myeloid lineage during chronic infection, and here, with the HIV-infected group in the current investigation.

The Th1 polarizing heterodimeric cytokine IL-12 induces IFN-γ secretion by T cells to augment the lytic activity of NK cells, and cytolytic T cells (70). The observed increase in plasma IL-12 across all the viral-infected groups although insignificant, reflects the host’s response against viral infection. Evidence also suggest that IL-12 response may be crucial in the development of hepatic damage in patients with chronic HBV infection, and hence in addition to aiding in viral clearance, this polarized profile may also contribute to the liver pathogenesis (71). Findings suggest that individuals with HCV infection have impaired cellular immunity, and IL-12 can salvage the responses in these patients (72). Further, plasma IL-12 could serve as a measure of cellular immunity to HBV infection (73). Increased IL-12 improves the antiviral functions of T cells in HBV infection (45). There is unambiguous agreement that better outcomes are related with increased levels of IL-12 in the peripheral blood of HBV-infected patients (46, 47).

Macrophage inflammatory protein-1 beta, also known as CCL4 binds CCR5 naturally to inhibit HIV fusion given that R5-tropic HIV-1 infects T cells *via* the CCR5 co-receptor (74). The high levels of MIP-1β seen among all the viral infection groups is suggestive of the magnitude of inflammation that may have implications, both protective or pathological. Further, in line with earlier research, we have reported herein that TNF-α levels predominate in HBV infection (26). Studies have revealed elevated plasma levels of IFN-γ and TNF-α in HBV infection (27) although protracted levels may likely promote neoplastic transformation (28). IL-8 (also called as CXCL8) is a neutrophil-recruitment factor that can protract the course of acute inflammation. CXCL8 has been linked to the emergence of more severe forms of chronic viral liver disorders, and their levels may be reflective of poor prognosis in HCV infection (37). Hence, downregulating IL-8 levels may be thought of as a potential strategy aimed at decreasing inflammation in chronic HIV infection (38).

Interleukin-6 is an endogenous pyrogen that together with IL-6 and TNF-α directs the liver to release acute-phase reactants during inflammation. IL-6 has substantially been linked with persistent HCV infection (39). The hyper-inflammatory IL-1β-enriched monocytes may be a key source of IL-6 and systemic inflammation among individuals with HIV infection (75). An HIV-infected patient cohort with alcoholism showed a substantial and significant correlation between IL-6 and liver fibrosis. IL-6 may be a helpful prognostic marker for liver fibrosis among individuals with HIV infection (76). It has been demonstrated by numerous studies that IL-6 can control viral entry and HBV replication. Besides, IL-6 also confers regenerative and protective properties and is key to onset and sustenance of inflammation (77). In our study Th1 cytokines had shown mild to moderate level of significance.

Interleukin-4 is down-regulated during chronic HBV infection in comparison to healthy controls (HC) and has an inverse relationship with virus load and HBsAg titers (34) although our study reports the reverse. Disease progression in chronic HBV infection leads to metabolic syndrome and chronic inflammation, with IL-13 playing a critical role in connecting the metabolic and inflammatory components (42). IL-5 plays a key pathogenic function in the differentiation, attraction, survival, and degranulation of eosinophils (29). IL-5 has been shown to play a crucial role in the immune response to challenge infection (78). Therefore, Th2 cytokines play a vital role in inflammatory responses as our study implies high level of IFN-γ, IL-4, IL-5, IL-13. We also found that IL-5 was inversely correlated with HBV DNA reflecting that IL-5 likely aids in controlling HBV viral load although further studies are warranted in affirming the downstream mechanisms of potential viral control. Under physiological and pathological conditions, IL-5 acts on a variety of target cells (79). IL-5 is a key component of the immune system’s defense against a variety of viral infections (80). The co-expression of viral antigen and IL-5 may alter antiviral immune responses. While replicating *in vitro*, SHIV appeared to benefit from co-expression of IL-5 in terms of proliferation. The insertion of IL-5 in a gene-deleted SHIV chimera boosts the immunogenicity (81). Higher plasma IL-5 concentrations were associated with successful ART treatment for HIV-associated KS (82).

Interleukin-10 and TNF-α are associated with substantial fibrosis in HBV infection, and may be involved in mediating the regulatory role of T cells (83). IL-10 in chronic HBV infection boosts Treg cells while suppressing HBV-specific CD4+ and CD8 + T cell responses (54). IL-10 has a favorable correlation with both hepatic flares and SGPT and SGOT levels (53). IL-17A are key biological indicators of immunopathogenesis in chronic hepatitis, and Th17-associated cytokines are linked to liver disease progression in HCV disease (41). Additionally, the frequency of IL-17-producing cells is higher in HIV-infected patients showing a positive correlation with indicators of baseline PMN activity (84). Hence, a positive increase in IL-17 encodes biological indication of liver damage in line with liver transaminases in the current study.

Given that HIV is an immune-compromising illness, CD4+ T cell count remains a key component in the monitoring of HIV disease progression following HAART (85). In agreement with the mechanisms described, our observation points to inverse correlation between HIV PVL and CD4+ T cell counts (86, 87). Our results are in line with the fact that HIV infection gradually results in immune dysfunction, which is pathognomonic of HIV disease progression. IFN-γ appears to be critical for advancing HIV disease pathogenesis (48) given its wide-ranging and profound impacts on immune activation, proinflammatory reactions, and immune regulation, as well as multiple other biological functions.

## Conclusion

In summary, we have characterized the predominant cytokines in chronic HBV, HCV, and HIV infections in association with viral loads and liver enzymes. These findings suggest a possible role of cytokines associated with disease severity, effective immune responses, and viral persistence, and serves as a key catalogue of biological responders of disease progression in chronic HBV, HCV, and HIV infections. IL-5 appears to play a key role in controlling viral replication likely aiding in fighting the HBV virus. A noteworthy increase in cytokines appears to profound the risk of disease progression and may likely bear protective and pathological significance, and indeed is reflective of the host’s versatile armamentaria against viral persistence.

## Data availability statement

The original contributions presented in this study are included in the article/Supplementary material, further inquiries can be directed to the corresponding authors.

## Ethics statement

The studies involving human participants were reviewed and approved by Institutional Ethical Committee (IEC) of the Government Medical College, Theni, India (Ref. Nos. 2544/ME1/18 and 1515/MEIII/21). The patients/participants provided their written informed consent to participate in this study.

## Author contributions

JV, YY, AM, and ES designed the study. JV, RA, and ES performed the experiments. AM, KV, VV, ML, and ES provided the regulatory oversight. ML and ES provided the project management. JV, YY, AM, ES, SR, and ML collected the study data and oversaw the participant visits. YY, ES, JV, MR, ED, and ML performed the participant data analysis and interpretation. JV, AM, and ES collected and analyzed the patient data and interpretation was done by YY, ES, ML, ED, VV, and PB. JV, YY, RA, and ES wrote the manuscript. JV, AM, MR, YY, ES, and ML accessed and verified the data. All authors contributed to the reviewing and editing of the report and approved the final version.

## Supplementary material

Supplementary Figure 1 Spearman correlation between CD4+ T cell counts and cytokines. *P*-values < 0.05 are considered significant in all tests; **p* < 0.05, ***p* < 0.01. HAART, highly active anti-retroviral therapy; HIV, human immunodeficiency virus; VL, viral load.
