# Supplementary material for: Plasma interleukin-7 correlation with human immunodeficiency virus RNA and CD4+ T cell counts, and interleukin-5 with circulating hepatitis B virus DNA may have implications in viral control
Source: Front Med (Lausanne). 2022 Nov 3;9:1019230. doi: 10.3389/fmed.2022.1019230 (PMC9668853; doi:10.3389/fmed.2022.1019230)
Supplement: Supplementary file 1 [file Table_2.DOCX]

**Supplementary Table**

| **Cytokine** | **Bead color code** | **Lowest detection limit (pg/ml)** |
| --- | --- | --- |
| G-CSF | 57 | 800 |
| GM-CSF | 34 | 41 |
| IFN-γ | 21 | 230 |
| IL-1β | 39 | 26 |
| IL-2 | 38 | 175 |
| IL-4 | 52 | 15 |
| IL-5 | 33 | 578 |
| IL-6 | 19 | 41 |
| IL-7 | 74 | 292 |
| IL-8 | 54 | 45 |
| IL-10 | 56 | 76 |
| IL-12 (p70) | 75 | 150 |
| IL-13 | 51 | 33 |
| IL-17 | 76 | 225 |
| MCP-1 | 53 | 48 |
| MIP-1β | 18 | 44 |
| TNF-α | 36 | 275 |
