# Supplementary figures and images for: Plasma interleukin-7 correlation with human immunodeficiency virus RNA and CD4+ T cell counts, and interleukin-5 with circulating hepatitis B virus DNA may have implications in viral control
Source: Front Med (Lausanne). 2022 Nov 3;9:1019230. doi: 10.3389/fmed.2022.1019230 (PMC9668853; doi:10.3389/fmed.2022.1019230)

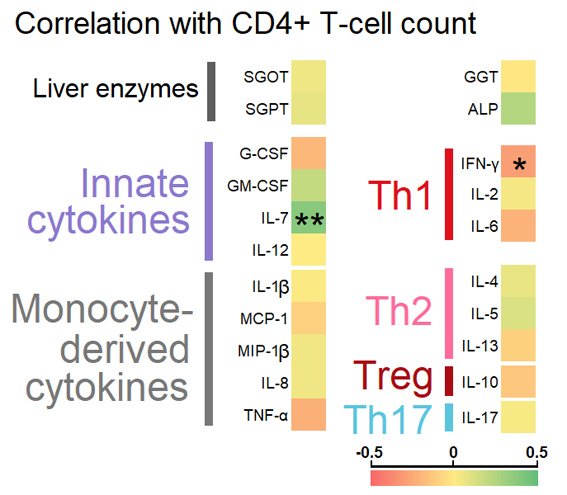

Supplement: Supplementary Figure 1 — Spearman correlation between CD4+ T cell counts and cytokines. P-values < 0.05 are considered significant in all tests; *p < 0.05, **p < 0.01. HAART, highly active anti-retroviral therapy; HIV, human immunodeficiency virus; VL, viral load. [file Image_1.PNG]
